# Development of a High-Throughput Serum Neutralization Test Using Recombinant Pestiviruses Possessing a Small Reporter Tag

**DOI:** 10.3390/pathogens9030188

**Published:** 2020-03-04

**Authors:** Madoka Tetsuo, Keita Matsuno, Tomokazu Tamura, Takasuke Fukuhara, Taksoo Kim, Masatoshi Okamatsu, Norbert Tautz, Yoshiharu Matsuura, Yoshihiro Sakoda

**Affiliations:** 1 Laboratory of Microbiology, Division of Disease Control, Faculty of Veterinary Medicine, Hokkaido University, Sapporo, Hokkaido 060-0818, Japan; doma2_1996@eis.hokudai.ac.jp (M.T.); matsuno@vetmed.hokudai.ac.jp (K.M.); tatatataksoo-u2@eis.hokudai.ac.jp (T.K.); okamatsu@vetmed.hokudai.ac.jp (M.O.); 2 Global Station for Zoonosis Control, Global Institute for Collaborative Research and Education (GI-CoRE), Hokkaido University, Sapporo 001-0020, Japan; 3 Department of Molecular Virology, Research Institute for Microbial Diseases, Osaka University, Osaka 565-0871, Japan; ttamura@princeton.edu (T.T.); fukut@biken.osaka-u.ac.jp (T.F.); matsuura@biken.osaka-u.ac.jp (Y.M.); 4 Department of Molecular Biology, Princeton University, Washington Road, Princeton, NJ 08540, USA; 5 Institute of Virology and Cell Biology, University of Lübeck, D-23562 Lübeck, Germany; norbert.tautz@vuz.uni-luebeck.de

**Keywords:** border disease, bovine viral diarrhea, classical swine fever, pestivirus, serum neutralization test, reporter virus

## Abstract

A serum neutralization test (SNT) is an essential method for the serological diagnosis of pestivirus infections, including classical swine fever, because of the cross reactivity of antibodies against pestiviruses and the non-quantitative properties of antibodies in an enzyme-linked immunosorbent assay. In conventional SNTs, an immunoperoxidase assay or observation of cytopathic effect after incubation for 3 to 7 days is needed to determine the SNT titer, which requires labor-intensive or time-consuming procedures. Therefore, a new SNT, based on the luciferase system and using classical swine fever virus, bovine viral diarrhea virus, and border disease virus possessing the 11-amino-acid subunit derived from NanoLuc luciferase was developed and evaluated; this approach enabled the rapid and easy determination of the SNT titer using a luminometer. In the new method, SNT titers can be determined tentatively at 2 days post-infection (dpi) and are comparable to those obtained by conventional SNTs at 3 or 4 dpi. In conclusion, the luciferase-based SNT can replace conventional SNTs as a high-throughput antibody test for pestivirus infections.

## 1. Introduction

Pestiviruses are enveloped positive-strand RNA viruses that belong to the genus *Pestivirus*, within the family *Flaviviridae*. Pestiviruses can infect farmed pigs and ruminants with significant economic impact and have also been detected in wild boar, wild ruminants, rodents, bats, and aquatic mammals [1,2]. Pestiviruses possess a single-stranded positive-sense RNA of approximately 12.3 kb in length, with one large open reading frame (ORF) flanked by 5’ and 3’ untranslated regions. The ORF encodes a single polyprotein cleaved by cellular and viral proteases co- and post-translationally into four structural proteins (C, E^rns^, E1, and E2) and eight non-structural proteins (N^pro^, p7, NS2, NS3, NS4A, NS4B, NS5A, and NS5B) [1]. The genus *Pestivirus* currently comprises 11 species, *Pestivirus A* to *Pestivirus K*, with bovine viral diarrhea virus (BVDV), classical swine fever virus (CSFV), and border disease virus (BDV) classified into *Pestivirus A* (BVDV-1), *Pestivirus B* (BVDV-2)*, Pestivirus C* (CSFV), and *Pestivirus D* (BDV), respectively [3]. Originally, the taxonomic classification of pestiviruses was based on the host species from which they were isolated (e.g., CSFV from pigs and BVDV from cattle), but it is now well known that many pestiviruses are capable of interspecies transmission (e.g., BVDV infections in pigs and BDV infections in cattle) [4,5].

Classical swine fever (CSF) is one of the most important diseases of domestic pigs and wild boar. Because of its tremendous impact on animal health and the pig industry, CSF is notifiable to the World Organization for Animal Health (OIE) [6,7,8]. The diagnosis of CSF consists of (1) clinical observation, (2) gross pathological findings, (3) antigen detection, and (4) antibody detection [9,10]. Diagnosis during the early stages of a CSF outbreak usually relies on 1 and 2 (i.e., clinical and pathological diagnoses), however, these features may vary and can sometimes be atypical [7,11,12]. Thus, for the confirmation of CSFV infection, antigen and antibody detection following the early clinical and pathological diagnoses is necessary. In the diagnostic laboratory, antigen detection by virus isolation and reverse transcriptase-polymerase chain reaction (RT-PCR) is highly recommended to confirm clinical cases. The detection of virus-specific antibodies is particularly useful for herds suspected of having been infected at least 21 days previously with CSFV [8]. Anti-CSFV antibody detection methods, such as enzyme-linked immunosorbent assay (ELISA), are valuable tools for surveillance that requires high-throughput, although this approach can be hampered by antibodies that cross-react with CSFV antigens, which can occasionally be raised in animals infected with other pestiviruses [13]. Some ELISAs are relatively CSFV-specific, but the definitive method of choice for differentiation is the comparative serum neutralization test (SNT), which compares the neutralizing titer of antibodies against different pestivirus isolates [8,14].

In September 2018, the first CSF outbreak in Japan for 26 years was reported [15,16]. Despite countermeasures being taken, including the culling of infected herds and movement restrictions, the infection has continued to spread in 10 prefectures, resulting in 57 outbreaks and a total of 165,186 pigs culled as of 2 March 2020 [17]. In addition, 1944 cases of CSFV infection in wild boar have been reported as of 21 February 2020 [17]. To control CSF in wild boar, a vaccination program using the bait dosed with vaccine containing a live attenuated C strain [18] was initiated in March 2019, in addition to efforts to reduce the wild boar population by trapping or hunting, based on previous experiences in Europe [19,20]. Furthermore, in addition to the improvements in biosecurity, a vaccination program using an injectable vaccine containing a live attenuated GPE^−^ strain [21] was also started in October 2019, to help minimize the CSF outbreak in domestic pigs. The vaccination of domestic pigs is only permitted in high-risk prefectures where CSFV infection in wild boar has been confirmed. Currently, large-scale serological monitoring is being conducted using ELISA, to evaluate the effects of the vaccination program and monitor the CSF-free status in non-vaccinated areas. In addition, BVDV and BDV infections in domestic pigs have also previously been reported in Japan [22,23]. Hence, the necessity for a comparative SNT is now increasing, both to discriminate CSFV-specific antibodies from those against BVDV or BDV and to understand quantitative aspects of antibody levels following the vaccination of wild boar and domestic pigs.

Despite the intense demands for the use of an SNT to test the sera of domestic pigs and wild boar, conventional SNTs based on an immunoperoxidase assay or cytopathic effect (CPE) observation are time- and labor-intensive when testing a large number of samples [8,24]. Thus, in this study, a new, high-throughput SNT method using recombinant viruses carrying a reporter gene was developed. Since the first recombinant pestivirus carrying a marker gene was constructed [25], various reporter genes have been applied for pestivirus research [26,27,28]. Recently, to reduce the risk of undesirable effects following the insertion of a large foreign gene into a viral genome, NanoLuc binary technology (NanoBiT) [29] was employed for the construction of recombinant CSFV and BVDV-1 carrying a small reporter gene [30,31]. NanoBiT is a split reporter protein consisting of two subunits: a high-affinity NanoBiT (HiBiT) consisting of just 11 amino acids and a large NanoBiT (LgBiT). Neither subunit has enzymatic activity individually but regains this activity when the subunits associate to form a heterodimer, either in cells or in vitro [32]. The detection of HiBiT-fusion viral protein is simple and scalable in comparison with the currently used methods.

In the present study, a recombinant BDV carrying the HiBiT gene was newly constructed and applied for SNT, together with previously established recombinant CSFV and BVDV-1 possessing HiBiT [30,31], to reduce the time and effort required to detect specific neutralizing antibodies. This new SNT method using these recombinant viruses showed the same sensitivity and specificity as conventional SNTs. The HiBiT recombinant-virus-based SNT will provide a more simple and rapid procedure for the serological diagnosis of pestivirus infections.

## 2. Results

### 2.1. Rescue of Border Disease Virus (BDV) Possessing a Small Reporter High-Affinity NanoBiT (HiBiT) Tag, vBDV FNK/HiBiT 

**Figure 1:**
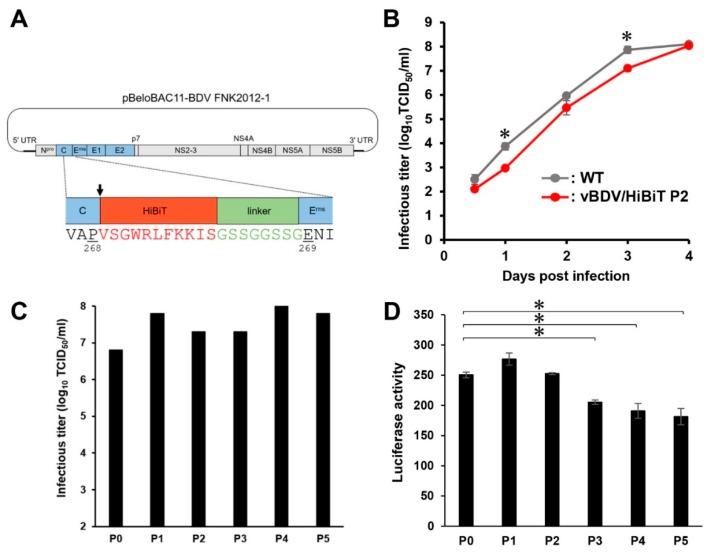
Construction and characterization of recombinant border disease virus (BDV) carrying high-affinity NanoBiT (HiBiT). (**A**) Schematic diagram of the BDV cDNA clone encoding the HiBiT luciferase gene. Amino acid sequence of HiBiT (red) and linker (green) was inserted upstream of E^rns^ of BDV FNK2012-1 strain (indicated by arrow). (**B**) Swine kidney line-L (SK-L) cells were infected with 200 50% tissue-culture infective doses (TCID_50_) of BDV FNK-WT (WT) and the recombinant vBDV FNK/HiBiT P2 (vBDV/HiBiT P2). Infectious titers in the culture supernatant were determined at the indicated time points. Data are presented as mean ± standard error (SE) (*n* = 3). Asterisks indicate significant differences (*, *p* < 0.05) versus the result of the vBDV/HiBiT P2. (**C**) SK-L cells were infected with 200 TCID_50_ of vBDV FNK/HiBiT, then the recombinant BDV was passaged five times. Undiluted culture supernatant (100 µL) at 3 days post-infection (dpi) was used for the next passage in naïve cells, and infectious titers in the culture supernatant were determined at 4 dpi after each passage. (**D**) SK-L cells were infected with 200 TCID_50_ of vBDV FNK/HiBiT, then the recombinant BDV was passaged five times. Luciferase activity in the cells was determined at 4 dpi after each passage. Data are presented as mean ± standard error (SE) (*n* = 3). Asterisks indicate significant differences (*, *p* < 0.05) versus the result of the vBDV FNK/HiBiT P0.

In our previous studies, recombinant *Flaviviridae* viruses possessing a small reporter HiBiT tag were developed as tools to understand the viral life cycle and pathogenesis and to provide a robust platform for the development of novel antivirals [30,31]. For those studies, two recombinant pestiviruses, vCSFV GPE^−^/HiBiT and vBVDV-1 NCP7/HiBiT, were established from the recombinant full-length cDNA of CSFV GPE^−^ and BVDV-1 NCP7 strains, respectively. To establish a novel SNT using pestiviruses possessing a small reporter tag, the recombinant full-length cDNA of the BDV FNK2012-1 strain, BDV subgenotype-1 (BDV-1) isolated from domestic pigs in Japan [22] was newly established and a BDV recombinant was generated by inserting HiBiT between C and E^rns^ (vBDV FNK/HiBiT, Figure 1A), since the N-termini of E^rns^ of CSFV and BVDV tolerate the exogenous tag [27,30,31]. To compare the growth of recombinant vBDV FNK/HiBiT with the wild-type BDV, swine kidney line-L (SK-L) cells were inoculated with 200 50% tissue-culture infective doses (TCID_50_) of either virus. The growth of the recombinant BDV was relatively lower at the early period, but finally identical to that of the wild-type BDV (Figure 1B). Next, the genetic stability of the newly constructed recombinant BDV was evaluated by passaging it at five times in SK-L cells. The infectious titer through the passages was sufficient (Figure 1C); however, the luciferase activities of vBDV FNK/HiBiT P3, P4 and P5 were significantly lower than that of original virus, vBDV FNK/HiBiT P0 (Figure 1D), suggesting low stability of this marker virus. For this reason, the serial passage of vBDV FNK/HiBiT for the SNT was kept to a minimum.

### 2.2. Characterization of Reporter Pestiviruses with HiBiT Tags

**Figure 2:**
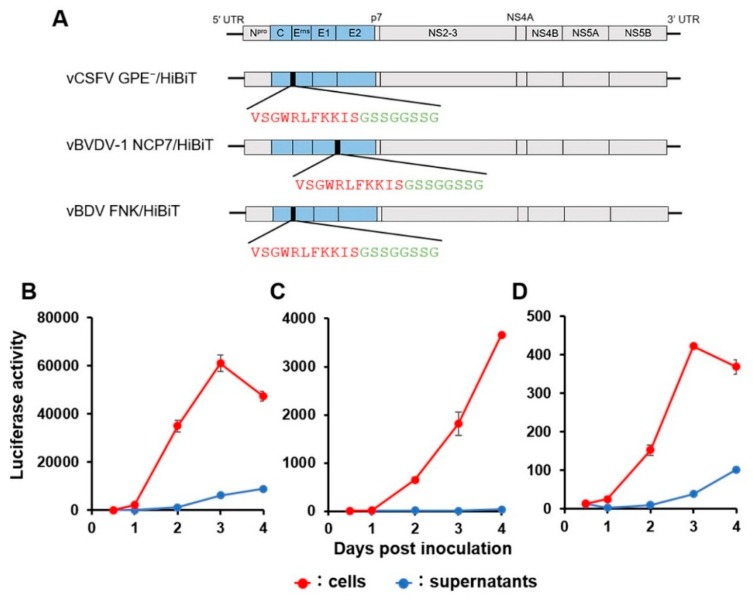
Construction and luciferase activity of the recombinant pestiviruses carrying HiBiT. Schematic preparation of the reporter pestiviruses (**A**). Coding regions of the recombinant viruses are displayed as boxes divided by each protein gene; structural protein (blue) and non-structural protein (grey). Amino acid sequence of HiBiT (red) and linker (green) was inserted upstream of E^rns^ of classical swine fever virus (CSFV) and BDV or upstream of E2 of BVDV-1. The luciferase activities following the infection of recombinant vCSFV GPE^−^/HiBiT in swine kidney line-L (SK-L) cells (**B**), vBVDV-1 NCP7/HiBiT in Madin–Darby bovine kidney (MDBK) cells (**C**), and vBDV FNK/HiBiT in SK-L cells (**D**) at 200 TCID_50_ were determined in the cell lysate or the culture supernatant, respectively. Data are presented as mean ± standard error (SE) (*n* = 3).

To check the increasing luciferase activity associated with virus growth, the luciferase activity in the infected cells and culture supernatant was measured during the time course of infection with vCSFV GPE^−^/HiBiT, vBVDV-1 NCP7/HiBiT, and vBDV FNK/HiBiT, respectively (Figure 2). Luciferase activity became detectable at 2 days post-infection (dpi) in the cells and the culture supernatant. For all infection experiments, the luminescence value in the cell lysate was higher than that of the respective culture supernatant. The luciferase activity in cells inoculated with vCSFV GPE^−^/HiBiT was the highest, followed by vBVDV-1 NCP7/HiBiT, while the lowest activity was measured for the cells inoculated with vBDV FNK/HiBiT. Taking these findings together, it was evident that the growth of each recombinant virus could be monitored based on luciferase activity in the cell lysate. The growth of vCSFV GPE^−^/HiBiT could also be monitored in culture supernatant.

To confirm whether the antigenicity of the recombinant viruses was affected by the insertion of HiBiT, both recombinants and parental viruses were tested using reference antisera in a conventional SNT based on the immunoperoxidase assay (Table 1). Convalescent sera derived from pigs experimentally infected with CSFV GPE^−^, BVDV-1 Nose, BVDV-2 KZ91, or BDV FNK2012-1 strains were used as reference antisera. These four pestiviruses are generally used as reference virus strains of pestiviruses for SNTs performed in Japan. In addition, the reference virus strain BVDV-1 Nose-WT was also tested, because the antiserum against BVDV-1 NCP7 was not available for this study. In the SNT using CSFV, the serum against CSFV showed the highest SNT titer, 1024, for both recombinant and wild-type viruses, while the other sera showed titers of <2. In the SNT using BVDV-1, however, the sera against BVDV-1 as well as BVDV-2 showed the highest SNT titer, 32, for the recombinant vBVDV-1 NCP7/HiBiT and the original vBVDV-1 NCP7-WT, while the other sera showed lower titers of <2. In terms of the neutralizing activities of antisera against BVDV-1 Nose and BVDV-2 KZ91-NCP to BVDV-1 Nose-WT, the serum against BVDV-1 Nose showed the highest SNT titer, 64, and the others against BVDV-2 KZ91-NCP showed lower titers of 8. In the SNT using BDV, serum against BDV showed the highest SNT titer, 128, for the recombinant and the wild-type viruses, and the other sera showed lower titers, of between 2 to 8. Each type of serum showed the highest SNT titer when the homologous virus was used, regardless of whether it was the wild-type virus or the recombinant virus, and there were few differences in SNT titer between the recombinant and wild-type viruses. Thus, the antigenicity of the recombinants was not altered by the insertion of HiBiT, although the BVDV-1 NCP7 strain with and without HiBiT showed different antigenicity compared with that of the BVDV-1 Nose strain. Hence, these recombinants carrying HiBiT were employed for the development of a new SNT.

### 2.3. Development of the Serum Neutralization Test (SNT) Based on the Luciferase Assay

The luciferase activity became detectable on 2 dpi for all viruses tested (Figure 2), therefore the new luciferase-based SNTs were first tested on 2, 3, and 4 dpi to determine the most appropriate incubation time for measuring luciferase activity (Supplementary Materials Figures S1–S3). Luciferase activity was monitored using the culture medium (for CSFV) or cell lysate (for BVDV-1 and BDV), because only CSFV luciferase activity in the culture medium was sufficient for the test. Luciferase activity increased depending on the serum dilution and the time following inoculation with the virus. At some of the low serum dilutions, luciferase activity was initially not detectable (e.g., vBDV FNK/HiBiT with the reference anti-BDV serum diluted 8 times, shown in Figure S3D), but became detectable by 4 dpi. On the other hand, at levels of serum dilution where the virus could be completely neutralized, the luciferase activity did not increase during the study period. Next, the SNT titers of the luciferase-based SNT were determined for each dpi and compared with the titers of the conventional SNT, determined on 4 dpi (Table 2). Following the luciferase-based assay from 2 to 4 dpi, each serum sample showed the highest SNT titer and 50% effective concentration (EC_50_) against the homologous strain and could be differentiated from the heterologous strains, with the exception of vBVDV-1 NCP7/HiBiT, which showed cross reactivity with the serum against BVDV-2. These findings were observed in both the luciferase-based and conventional SNT, with SNT titers based on luciferase activity decreasing in a time-dependent manner and finally becoming comparable with SNT titers from the conventional SNT at 3 or 4 dpi.

## 3. Discussion

ELISAs involve simple, rapid procedures and are therefore widely used for the serological diagnosis of pestivirus infections, including CSF. Because of the cross reactivity of antibodies against pestiviruses, the definitive method of choice for differentiation of pestiviruses is the comparative SNT, which compares neutralizing titers of antibodies to different pestivirus isolates [8]. However, the conventional SNT requires a high-containment laboratory able to handle infectious viruses and involves labor-and time-intensive procedures. Therefore, recombinant pestiviruses were employed to establish a novel SNT based on the luciferase system, which allows virus growth to be easily and rapidly monitored and enables SNT titers for pestiviruses to be determined.

Four pestiviruses, including CSFV, BVDV-1, BVDV-2, and BDV, are usually used as reference viruses for SNTs. The CSFV GPE^−^ strain, which shows a CPE in CPK-NS cells [24], the BVDV-1 Nose and BVDV-2 KZ91-CP strains, which show a CPE regardless of the cell line used, and the BDV FNK2012-1 strain, which does not show a CPE, are usually selected for conventional comparative SNTs in Japan. In SNTs using reference viruses which do not show a CPE, the immunoperoxidase assay is performed to determine the SNT titers following incubation for 3 to 4 days, which is indicated in the OIE manual to be the “gold standard” [8], even though it is time-consuming. In the present study, SNT titers determined by a luciferase-based SNT using recombinant pestiviruses were comparable with those determined using conventional SNTs, although some differences in SNT titers were observed; this might reflect the experimental conditions, such as the type of cells or viruses used. The luciferase assay used to determine SNT titers is less time-consuming and more straightforward than the immunoperoxidase assay when dealing with a large number of samples, although the recombinant pestiviruses can only be handled by a limited number of laboratories. In addition, the SNT titers can be determined within 3 or 4 days of inoculation, which is the same as the conventional SNT based on the immunoperoxidase assay and earlier than the conventional SNT based on CPE observation. Additionally, the SNT titers can be tentatively determined earlier, at 2 dpi, although they are slightly higher than those at 4 dpi, suggesting that earlier confirmation of pestivirus infection may be possible using the present method of SNT. 

In Japan, CSFV infection has spread among domestic pigs and wild boar since the first outbreak at a domestic pig farm in September 2018. Control of CSFV infection in wild boar should be prioritized for preventing the spread of CSF, because direct or indirect contact with wild boar infected with CSFV can be a primary route for the introduction of the virus to pig farms [33,34]. To prevent the spread of this infection in wild boar, an oral vaccination program targeting wild boar commenced in the spring of 2019. So far, the efficacy of vaccination in wild boar has been evaluated by ELISA only, while the cross-reaction to antibodies against other ruminant pestiviruses and non-specific reactions to the low-quality sera derived from wild boar have not been investigated. Hence, SNTs should be performed at reference laboratories to confirm the ELISA results and possibly also provide information about the spread of CSFV in the field. In addition to the vaccination of wild boar, a vaccination program for domestic pigs was re-started in October 2019. The vaccine being used in the present program is the same as that used in the previous program conducted in Japan between 1969 and 2006, in which domestic piglets aged from 30 to 40 days were vaccinated once [35]. Thus, in this program, in which it is recommended that all pigs in the at-risk area are vaccinated regardless of their age, the vaccine performance should be revisited according to the neutralizing antibody titer derived from acquired and/or maternal immunity. Hence, the SNT titer after the vaccination of domestic pigs, in addition to wild boar, should be investigated using this luciferase-based SNT.

**Figure 3:**
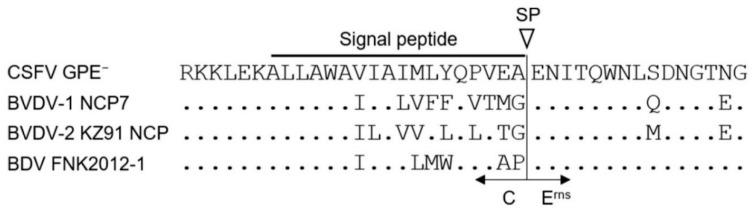
Amino acid sequences of pestiviruses around the cleavage site between C and E^rns^. The sequences were compared using GENETIX network ver. 12.0.1 (GENETYX Corp., Japan). The region of the signal peptide sequence and the site of cleavage by signal peptidase is indicated on top of the alignment (SP). The line and arrow indicate part of the C and E^rns^ proteins, respectively, at the bottom of the alignment.

The N-termini of E^rns^ of both CSFV and BVDV are capable of expressing an exogenous tag [27,30,31]. Based on these findings, we generated a BDV recombinant by inserting HiBiT between C and E^rns^ (vBDV FNK/HiBiT) (Figure 1A). However, the luciferase activity of vBDV FNK/HiBiT was lower than those of the others (Figure 2). In addition, the luciferase activities of serially passaged recombinant virus were significantly lower than that of original virus (Figure 1D); suggesting low genetic stability of the vBDV FNK/HiBiT. The luciferase activity of recombinant virus carrying HiBiT should be related to the expression level of HiBiT fused with a viral protein; therefore, HiBiT inserted into recombinant BDV might not be appropriately expressed, resulting in its lower luciferase activity. The vBDV FNK/HiBiT was designed following vCSFV GPE^−^/HiBiT to express HiBiT and the linker fused with the N-terminus of E^rns^ and presumably cleaved upstream of HiBiT by a signal peptidase. However, the signal sequence at the C-terminus of the C is not conserved among the pestiviruses (Figure 3); therefore, HiBiT inserted downstream of the signal sequence might disturb the post-translational cleavage of the BDV protein. Indeed, the growth of vBVDV-1 NCP7/HiBiT and vBDV FNK/HiBiT needs to be monitored by measuring luciferase activity in the cells, whereas vCSFV GPE^−^/HiBiT is able to be monitored by measuring luciferase activity in the culture supernatant (Figure 2). Measuring luciferase activity in the culture supernatant enables the assessment of viral growth at several time points after infection of a single cell culture. Thus, recombinant viruses need to be detectable in culture supernatants for an assay to be efficient. On the other hand, we evaluated vBVDV-1 NCP7/HiBiT, which has been previously reported [30,31], but the significant cross reactivity of vBVDV-1 NCP7-WT with the serum against BVDV-2 was confirmed. In addition, the subgenotypes of BDV are more diverse, and selection of a reference strain for the SNT seems to be more complicated, although only BDV subgenotype 1 (BDV-1) has previously been isolated in Japan [22]. Hence, appropriate reference viruses should be selected based on the local prevalence of pestiviruses, and genetically stable recombinant viruses, which express the HiBiT fused with a viral protein efficiently, should be generated for the luciferase-based SNT. These localized recombinant reporter pestiviruses would be useful for luciferase-based SNTs for the surveillance not only of CSFV but also, in certain areas, of BVDV and BDV.

Altogether, the present luciferase-based SNT enables SNT titers to be determined simply and rapidly, replacing conventional SNTs. Therefore, this luciferase-based SNT could be a powerful tool for high-throughput serological testing, providing useful information for the investigation and control of pestiviruses, including CSFV in the field.

## 4. Materials and Methods 

### 4.1. Cells and Viruses

The SK-L and MDBK cells were propagated in Eagle’s Minimum Essential Medium (Nissui Pharmaceutical, Tokyo, Japan) supplemented with 0.3 mg/mL L-glutamine (Nacalai tesque, Kyoto, Japan), 100 U/mg penicillin G (Meiji Seika Pharma, Tokyo, Japan), 8 µg/mL gentamycin (TAKATA Pharmaceutical, Saitama, Japan), sodium bicarbonate (Nacalai tesque), 0.1 mg/mL streptomycin (Meiji Seika Pharma), 0.295% tryptose phosphate broth (Becton, Dickinson and Company, Franklin Lakes, NJ, USA), 10 mM N,N-bis-(2-hydroxyethyl)-2-aminoethanesulfonic acid (BES; MilliporeSigma, St. Louis, MO, USA), and 10% horse serum (Thermo Fisher Scientific, Waltham, MA, USA). Both types of cells were incubated at 37 °C in the presence of 5% CO_2_.

Three recombinant pestiviruses encoding the HiBiT luciferase gene were used for the new luciferase-based SNT; vCSFV GPE^−^/HiBiT was derived from recombinant full-length cDNA of the CSFV GPE^−^ strain, which expressed HiBiT luciferase fused with the N-terminus of viral E^rns^ [31]. vBVDV-1 NCP7/HiBiT was derived from recombinant full-length cDNA of the BVDV-1 NCP7 strain, which expressed HiBiT luciferase fused with the N-terminus of viral E2 [30,36]. vBDV FNK/HiBiT was derived from recombinant full-length cDNA of the BDV-1 FNK2012-1 strain [22], which expressed HiBiT luciferase fused with the N-terminus of viral E^rns^. This full-length cDNA of the BDV FNK2012-1 strain and vBDV FNK/HiBiT was newly developed for this study. Details of the construction of the recombinant full-length cDNA clone of BDV are given below.

In addition, the wild-type viruses in the conventional SNT were selected as follows: vGPE^−^, which was derived from recombinant full-length cDNA of the CSFV GPE^−^ strain [37], described as vCSFV GPE^−^-WT, BVDV-1 Nose strain [38] described as BVDV-Nose-WT, vNCP7 which was derived from the recombinant full-length cDNA of the BVDV-1 NCP7 strain [36] described as vBVDV-1 NCP7-WT, and BDV FNK2012-1 strain [22] described as BDV FNK-WT. The BDV FNK2012-1 strain was isolated from domestic pigs and propagated in SK-L cells.

### 4.2. Reference Antisera

Four reference porcine sera were used for the SNTs. Each serum sample was obtained from a pig in the convalescent phase following infection with CSFV GPE^−^ strain, BVDV-1 Nose strain, BVDV-2 KZ91-NCP strain, or BDV FNK2012-1 strain. CSFV GPE^−^ strain was intramuscularly inoculated in 6-week-old pigs, and serum against CSFV was collected at 6 months post-inoculation. BDV FNK2012-1 strain was intramuscularly inoculated in four-week-old pigs, followed by intramuscular inoculation with adjuvant; serum against BDV was collected at 42 dpi. Porcine sera against BVDV-1 Nose strain and BVDV-2 KZ91-CP strain were previously established [14]. All sera were inactivated by heating at 56 °C for 30 min before SNTs were performed.

### 4.3. Construction of BDV FNK2012-1 Full-Length cDNA

The cDNA clone of BDV FNK2012-1 was constructed according to a previously reported methodology [39]. Viral RNA was extracted from the supernatant of virus-infected cells using TRIzol LS Reagent (Thermo Fisher Scientific), 1-bromo-3-chloropropane (MilliporeSigma), and RNeasy Mini Kit (Qiagen, Hilden, Germany), according to the manufacturer’s instructions. The extracted RNA was reverse transcribed (RT) with RT primer (Table S1) using SuperScript III Reverse Transcriptase (Thermo Fisher Scientific), and subsequently 2 µL of cDNA was subjected to polymerase chain reaction (PCR) with gene-specific primers (Table S1) using Q5 Hot Start High-Fidelity DNA Polymerase (New England Biolabs, Ipswich, MA, USA). The linearized fragment of pBeloBAC11 (New England Biolabs) was also amplified with the primers indicated in Table S1, using Q5 Hot Start High-Fidelity DNA Polymerase. Then, the bacterial artificial chromosome (BAC) containing full-length cDNA corresponding to FNK2012-1 was obtained using an In-Fusion HD Cloning Kit (TaKaRa Bio, Shiga, Japan). The nucleotide sequences of the purified PCR products of the cDNA clones used in the present study were confirmed using an ABI 3500 Genetic Analyzer (Thermo Fisher Scientific).

### 4.4. Construction of HiBiT Recombinant Full-Length cDNA of BDV

The BDV cDNA clone encoding the HiBiT luciferase gene (Figure 1) was constructed following the method described in a previous report for CSFV and BVDV-1 [30,31], using a KOD-plus-mutagenesis kit (TOYOBO, Osaka, Japan) and the respective oligonucleotide primers. Briefly, the BAC carrying the BDV cDNA clone was used as a template for inverse PCR following DpnI digestion, with primers including the HiBiT sequence (Table S1), and then the product was self-ligated, according to the manufacturer’s protocol.

### 4.5. In Vitro Transcription and RNA Transfection

The recombinant clones of BVDV-1 and BDV were used as templates for full genome PCR to add the T7 promoter sequence with the respective primers (Table S1), using AccuPrime *Taq* DNA Polymerase, High Fidelity (Thermo Fisher Scientific), and then the products were purified using a FastGene Gel/PCR Extraction Kit (NIPPON Genetics, Tokyo, Japan), according to the manufacturer’s protocol. The recombinant full-length cDNA clone of CSFV was linearized at the SrfI site located at the end of the viral genomic cDNA sequence, followed by purification by phenol-chloroform extraction and ethanol precipitation. The purified PCR products of the BVDV-1 and BDV clones and the linearized product of the CSFV clones were used as templates for run-off transcription using a MEGAscript T7 kit (Thermo Fisher Scientific). After DNase I digestion and purification in S-400 HR Sephadex columns (GE Healthcare, Chicago, IL, USA), RNA was transfected to SK-L cells for CSFV and BDV or MDBK cells for BVDV-1 by electroporation using a Gene Pulser Xcell (Bio-Rad, Hercules, CA, USA), set at 200 V and 500 µF for SK-L cells or 180 V and 950 µF for MDBK cells, followed by incubation at 37 °C for 3 days. Virus recovery was confirmed by an immunostaining assay using anti-CSFV NS3 antibodies, as described below. The entire genomes of rescued viruses were verified by sequencing with an ABI 3500 Genetic Analyzer. The rescued viruses were stored at −80 °C for the SNTs.

### 4.6. Immunoperoxidase Assay to Detect Pestivirus Antigens

The immunoperoxidase assay was performed as previously described [40]. Briefly, cells inoculated with viruses were washed with PBS and heat-fixed at 80 °C for 1 h. The cells were then incubated at room temperature for 1 h in the presence of the primary monoclonal antibody for NS3, 46/1. The cells were washed with PBS and then incubated at 37 °C for 1 h in the presence of goat anti-mouse IgG (H+L) horseradish peroxidase conjugate (Bio-Rad). The cells were washed again and then stained with 3-amino-9-ethyl carbazole (MilliporeSigma).

### 4.7. Virus Titration

SK-L cells were infected with 10-fold serially diluted CSFV or BDV, and MDBK cells were infected with 10-fold serially diluted BVDV-1, in 96-well plates and incubated at 37 °C for 4 days. Then, the plates were immunostained, as described above. Virus titers were calculated and expressed as TCID_50_ per mL [41].

### 4.8. Luciferase Assay

The luciferase activity of culture supernatants or cell lysates was measured using a Nano-Glo HiBiT lytic detection system (Promega, Madison, WI, USA), according to the manufacturer’s protocol. To measure luciferase activity in the cell culture supernatant, 20 µL of culture medium was mixed with an equal volume of Nano-Glo HiBiT lytic buffer. To measure luciferase activity in cell lysate, all culture medium was removed, and the cells were lysed using 20 µL of Nano-Glo HiBiT lytic buffer for 20 min at room temperature. Luciferase activity was measured in a 96-well LumiNunc^TM^ plate (Thermo Fisher Scientific) using a POWERSCAN 4 (DS Pharma Biomedical, Osaka, Japan).

### 4.9. Serum Neutralization Tests (SNTs)

Most of the SNTs were performed according to previously described protocols [8,14]. Equal volumes (25 µL) of serially diluted serum and 200 TCID_50_ of CSFV, BVDV-1, or BDV were mixed and incubated at 37 °C for 1 h. This mixture plus SK-L or MDBK cell suspension was incubated in 96-well plates at 37 °C and 5% CO_2_. Viral antigens were detected at 4 dpi using the conventional SNT by immunostaining assay, as described above.

For the new luciferase-based SNT, viral antigens were detected from the culture medium (for CSFV) or cell lysate (for BVDV-1 and BDV) on each day by the luciferase assay, as described above. The cut-off value for the luciferase activity to signal complete neutralization (i.e., no virus growth) for the new SNT was calculated based on that of mock-infected 96-well plates. The average number of mock-infected 96-well plates plus five times the standard deviation of this population (i.e., luciferase activity = 70) was set as the cut-off value.

The neutralizing antibody titer of each test was expressed as the reciprocal of the highest serum dilution which showed complete neutralization of the virus.

### 4.10. The 50% Effective Concentration (EC_50_) of Each Serum against Marker Virus

For the calculation of EC_50_, the diagram of obtained luciferase activity was figured out as a sigmoid curve using ImageJ (version 1.52t) [42] Then, the EC_50_ was calculated based on the maximum and minimum value of each test.

### 4.11. Ethics Statement

The animal experiments were authorized by the Institutional Animal Care and Use Committee of the Faculty of Veterinary Medicine, Hokkaido University (approval number 18-0038), and performed according to the guidelines of this committee.

## Figures and Tables

**Table 1:** Comparative serum neutralization tests (SNTs) of pestiviruses based on the conventional immunoperoxidase assay.

Marker Virus	SNT Titer* of Antiserum against			
	CSFV GPE^−^	BVDV-1 Nose	BVDV-2 KZ91-NCP	BDV FNK2012-1
vCSFV GPE^−^/HiBiT	1024	<2	<2	<2
vCSFV GPE^−^-WT	1024	<2	<2	<2
vBVDV-1 NCP7/HiBiT	<2	32	32	<2
vBVDV-1 NCP7-WT	<2	32	32	<2
BVDV-1 Nose-WT	<2	64	8	<2
vBDV FNK/HiBiT	8	2	4	128
BDV FNK-WT	8	4	8	128

* SNT titers were determined at 4 days post-infection (dpi).

**Table 2:** Comparative SNTs of pestiviruses when using the new luciferase-based assay and the conventional immunoperoxidase assay.

Marker Virus	SNT Method (dpi)		SNT Titer and EC_50_* of Antiserum against							
			CSFV GPE^−^		BVDV-1 Nose		BVDV-2 KZ91-NCP		BDV FNK2012-1	
vCSFV GPE^−^/HiBIT	Luciferase-based	(2)	1024	11.3	2	6.73	2	7.04	4	5.19
		(3)	1024	11.1	<2	2.46	<2	4.59	<2	3.65
		(4)	1024	10.8	<2	1.65	<2	3.75	<2	1.86
	Immunoperoxidase	(4)	1024		<2		<2		<2	
vBVDV-1 NCP7/HiBiT	Luciferase-based	(2)	2	4.98	128	8.47	128	9.57	<2	2.77
		(3)	<2	3.97	32	8.17	32	7.97	<2	2.01
		(4)	<2	2.93	32	7.91	64	9.52	<2	N/A
	Immunoperoxidase	(4)	<2		32		32		<2	
vBDV FNK/HiBiT	Luciferase-based	(2)	64	7.36	8	4.92	64	7.20	2048	10.83
		(3)	8	4.77	2	2.22	2	3.34	256	9.01
		(4)	2	1.01	<2	N/A	<2	1.22	128	7.11
	Immunoperoxidase	(4)	8		2		4		128	

* The EC_50_ number of the Luciferase-based SNT method was indicated by an index with a base of 2. N/A: not available.

## Supplementary Materials

The following are available online at https://www.mdpi.com/2076-0817/9/3/188/s1: Figure S1: Serum neutralization test (SNT) using recombinant CSFV carrying HiBiT and anti-pestivirus serum; Figure S2: SNT using recombinant BVDV-1 carrying HiBiT and anti-pestivirus serum; Figure S3: SNT using recombinant BDV carrying HiBiT and anti-pestivirus serum, Table S1: Primers for the construction of HiBiT recombinant full-length cDNA of BDV and the virus rescue for BVDV-1 and BDV.
