# Supplementary material for: Development of a High-Throughput Serum Neutralization Test Using Recombinant Pestiviruses Possessing a Small Reporter Tag
Source: Pathogens. 2020 Mar 4;9(3):188. doi: 10.3390/pathogens9030188 (PMC7157198; doi:10.3390/pathogens9030188)
Supplement: Supplementary file 1 [file pathogens-09-00188-s001.pdf]

Table S1. Primers for the construction of HiBiT recombinant full-length cDNA of BDV and virus rescue for BVDV-1 and BDV.

Primer	Direction [a]	Sequence (5′→3′) [b]
BDV-FNK-RT	R	GGGCTGTTAGGGTTTTTCCTTAATCCAA
BDV-FNK-F	F	GTATACGGGGTAGCTCATGCCCGTGTACAAAATTGGACTTT
BDV-FNK-R	R	GGGCTGTTAGGGTTTTTCCTTAATCCAACTCTGGACTTCA
BDV-FNK-15_pBR	F	AGCTACCCCGTATACGCGGCCGCCCGTCGACCAATTCTCATGTTTGACAGCTTATC
BDV-FNK-15_pBF	R	AAACCCTAACAGCCCGCGGCCGCATCGAATATAACTTCGT
FNK-Erns-HiBiT- F	F	attagcgggagttctggcggctcgagcggtGAGAATATAACACAATGGAACC
FNK-Erns-HiBiT- R	R	cttcttgaacagccgccagccgctcacTGGTGCTACAGGCTGCCACATCAG
BDV-T7-F	F	<u>TAATACGACTCACTATA</u>GTATACGGGGTAGCTCATGCCCGTGTACAAAATTGGACTTT
NCP-T7-F	F	<u>TAATACGACTCACTATA</u>GTATACGAGGTTAGGCAAGTTCTCGTATACATATTGGACAC
NCP-7-R	R	GGGGCTGTTAAGGGTTTTCCCTAGTCCAACC

[a] "F" indicates forward, and "R" indicates reverse.
[b] The underlined sequence indicates the T7 promoter sequence. Lower-case letters indicate the HiBiT and linker sequences.

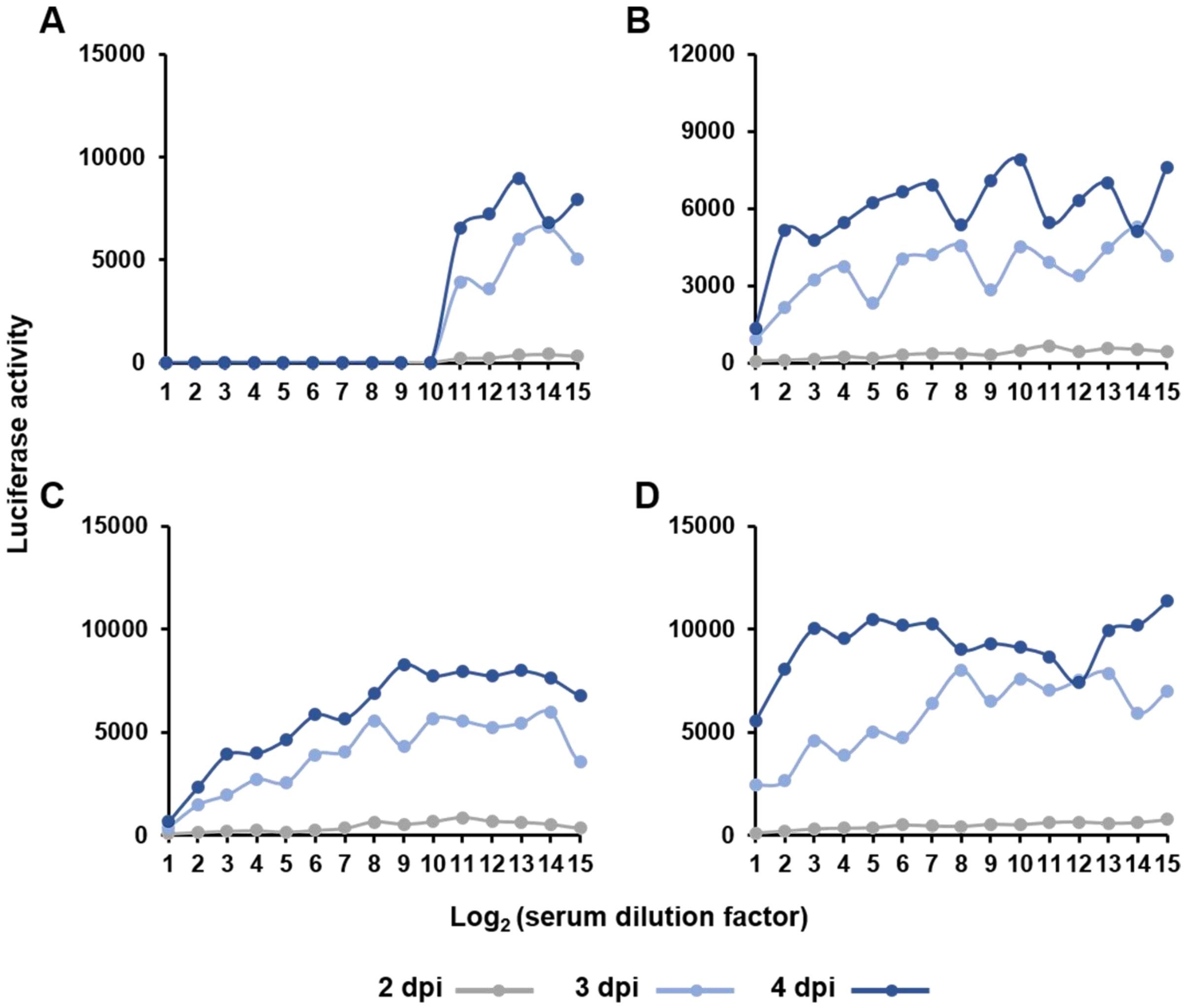

Figure S1. Serum neutralization test using recombinant CSFV carrying HiBiT and anti-pestivirus serum. vCSFV GPE⁻/HiBIT was incubated with two-fold serially diluted anti-CSFV serum (**A**), anti-BVDV-1 serum (**B**), anti-BVDV-2 serum (**C**), or anti-BDV serum (**D**). Luciferase activity in the culture supernatant of 96-well plates was determined on 2, 3, and 4 days post-infection (dpi). The cut-off value (luciferase activity = 70) to signal complete neutralization was calculated based on that of mock-infected 96-well plates as described in the Materials and Methods. The diagram of obtained luciferase activity was figured out as a sigmoid curve using ImageJ (version 1.52t) for the calculation of 50% effective concentration (EC_{50}).

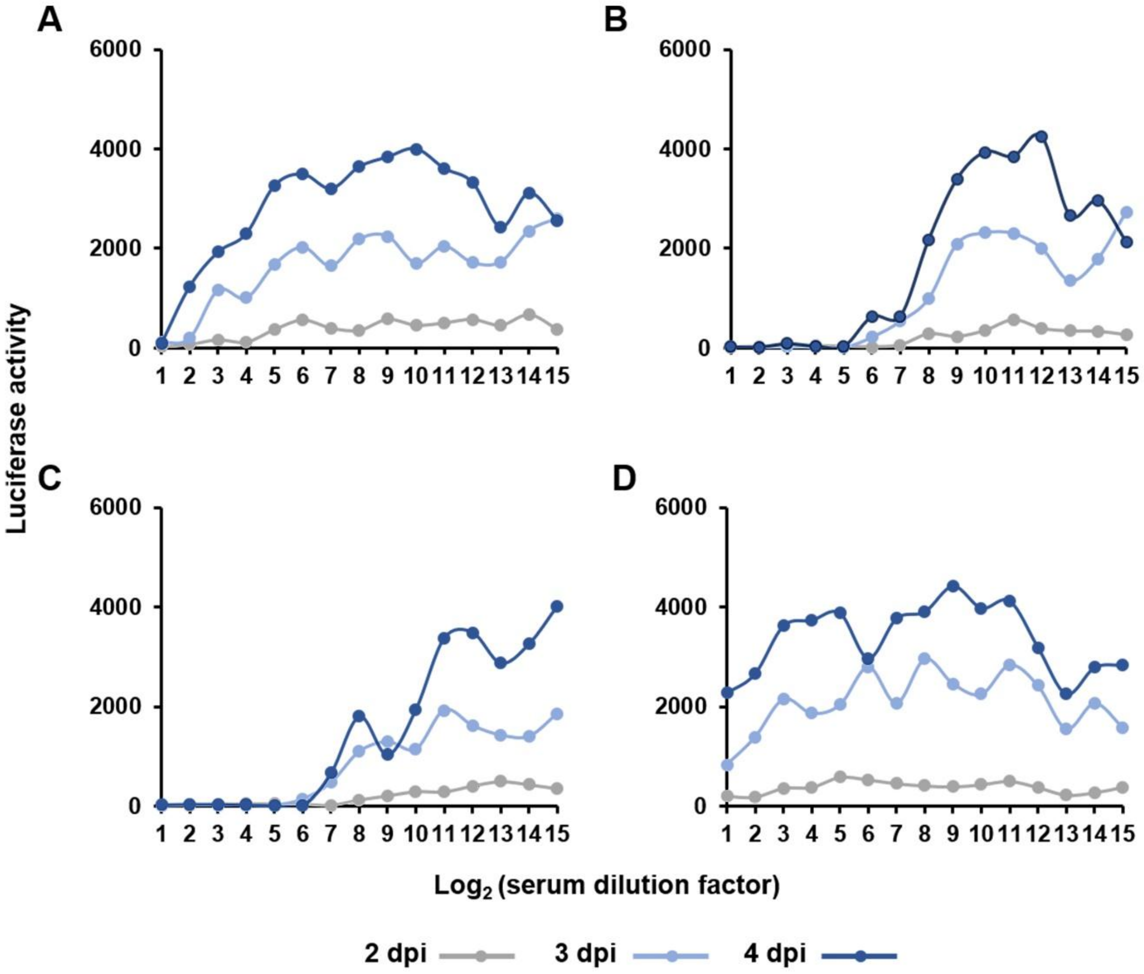

Figure S2. Serum neutralization test using recombinant BVDV-1 carrying HiBiT and anti-pestivirus serum. vBVDV-1 NCP7/HiBIT was incubated with two-fold serially diluted anti-CSFV serum (**A**), anti-BVDV-1 serum (**B**), anti-BVDV-2 serum (**C**), or anti-BDV serum (**D**). Luciferase activity in the cell lysate of 96-well plates was determined on 2, 3, and 4 days post-infection (dpi). The cut-off value (luciferase activity = 70) to signal complete neutralization was calculated based on that of mock-infected 96-well plates as described in the Materials and Methods. The diagram of obtained luciferase activity was figured out as a sigmoid curve using ImageJ (version 1.52t) for the calculation of 50% effective concentration (EC_{50}).

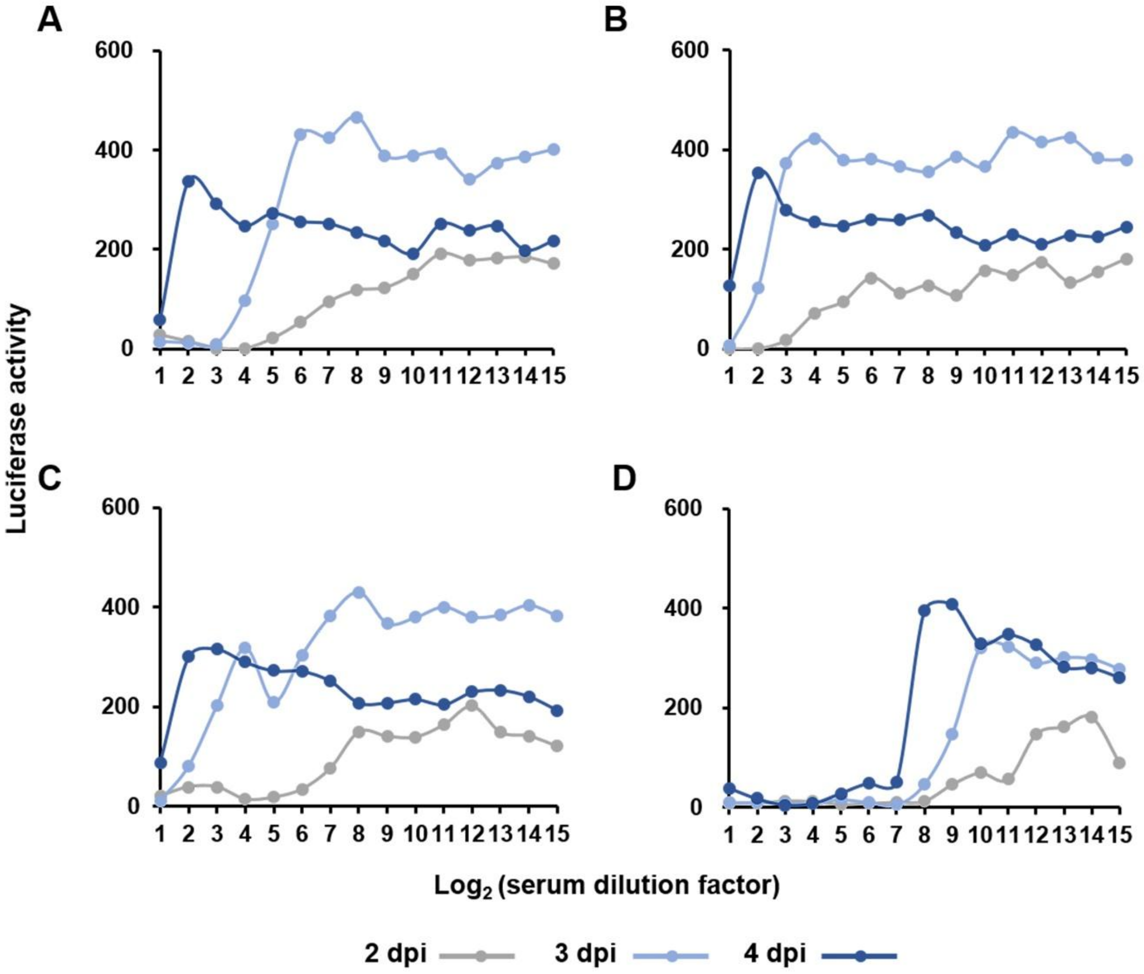

Figure S3. Serum neutralization test using recombinant BDV carrying HiBiT and anti-pestivirus serum. vBDV FNK/HiBIT was incubated with two-fold serially diluted anti-CSFV serum (**A**), anti-BVDV-1 serum (**B**), anti-BVDV-2 serum (**C**), or anti-BDV serum (**D**). Luciferase activity in the cell lysate of 96-well plates was determined on 2, 3, and 4 days post-infection (dpi). The cut-off value (luciferase activity = 70) to signal complete neutralization was calculated based on that of mock-infected 96-well plates as described in the Materials and Methods. The diagram of obtained luciferase activity was figured out as a sigmoid curve using ImageJ (version 1.52t) for the calculation of 50% effective concentration (EC_{50}).
